# Flax Lignans: From Biosynthetic Regulation to Biological Activities

**DOI:** 10.3390/ijms27167162

**Published:** 2026-08-11

**Authors:** Cheng Wang, Xiaofang Wang, Xin Liu, Zitong Li, Xinwu Pei, Yan Long

**Affiliations:** 1 Research Institute of Biology and Agriculture, School of Advanced Agricultural Sciences, University of Science and Technology Beijing, Beijing 100083, China; d202310500@xs.ustb.edu.cn (C.W.); w15737347613@163.com (X.W.);; 2 College of Life Science and Technology, Wuhan Polytechnic University, Wuhan 430023, China; 3 Biotechnology Research Institute, Chinese Academy of Agricultural Sciences, Beijing 100081, China

**Keywords:** flax lignans, biosynthesis, metabolic conversion, biological activities, extraction and purification

## Abstract

Flax lignans are abundant plant secondary metabolites in flaxseed, with secoisolariciresinol diglucoside (SDG) content reaching 0.61–1.33% of seed dry weight—substantially higher than levels reported for sesame seeds (0.01–0.03%), cereals (<0.01%), and most legumes (<0.005%). These levels make them natural bioactive compounds with considerable application potential. This review summarizes the biosynthetic pathways, key enzymes, and regulatory mechanisms of flax lignans; describes their major biological activities, including antioxidant, anti-inflammatory, anticancer, cardiovascular protective, and metabolic regulatory effects and possible mechanisms of action; and analyzes current progress in extraction, purification, analytical detection, and application. Key limitations in industrial application include complex extraction procedures, high production costs, lack of unified quality standards, and insufficient clinical evidence. Advanced strategies such as synthetic biology approaches for heterologous production, molecular breeding for high-lignan varieties, and green extraction technologies are discussed as promising solutions. This review is intended to serve as a conceptual reference for subsequent mechanistic studies, product development, and industrial translation of flax lignans in food, pharmaceutical, and cosmetic applications.

## 1. Introduction

Flax lignans are a class of plant lignans enriched in flaxseed. They are mainly polyphenolic secondary metabolites formed through oxidative coupling of phenylpropanoid units. Their content in flaxseed can reach 0.61–1.33%, making flaxseed an important recognized dietary source of lignans; these levels are generally higher than those in sesame seeds, cereals, and most legumes [1,2,3]. Among these compounds, secoisolariciresinol (SECO) is an important precursor, whereas secoisolariciresinol diglucoside (SDG) is the predominant lignan in flaxseed and commonly occurs in glycosylated forms and macromolecular complexes [4]. Flax is a major oilseed and fiber crop, and its seeds are rich in lignans such as SDG. Because flax lignans have a defined botanical source, broad dietary use, and potential health-related effects, they have attracted increasing attention in food nutrition, natural products, and bioactive-compound research [5]. This review provides a comprehensive and up-to-date synthesis of flax lignan research by integrating recent advances in biosynthetic regulation, biological activities, and industrial applications. Unlike previous reviews that focused on individual aspects, this work offers a systematic perspective from biosynthesis to application, highlighting the connections between regulatory mechanisms and functional properties and critically analyzing the gaps and future directions for industrial translation.

With respect to biosynthesis, existing studies indicate that flax lignan biosynthesis is closely associated with the phenylpropanoid pathway. The core biosynthetic route has been preliminarily clarified: L-phenylalanine serves as an upstream precursor and is converted through the phenylpropanoid pathway into monolignol intermediates, such as coniferyl alcohol, which are further catalyzed and modified by multiple enzymes to form major flax lignans such as SDG [6]. Key enzymes of the phenylpropanoid metabolism and the flax lignan biosynthetic pathway have been identified and functionally validated. Studies have clarified the position and regulatory roles of these enzymes in the biosynthetic pathway and demonstrated the close relationship between the activities of key enzymes and the biosynthesis of flax lignans [5,7,8]. In addition, transcriptomic, metabolomic and genetic transformation approaches have been advanced in the research of regulatory mechanisms of flax lignan biosynthesis. Accumulation of SDG and SECO is regulated by hormone signaling and transcription factors, as evidenced by current studies [9,10]. However, the overall biosynthetic regulatory network and the mechanisms by which environmental factors interact with biosynthetic pathways remain insufficiently understood.

In terms of biological activities, research on flax lignans mainly concerns the antioxidant activity, anti-inflammatory activity and regulation of lipid metabolism. However, most of the current evidence is still derived from in vitro experiments and animal models, whereas human studies are still relatively scarce [4]. Previous studies have shown that flax lignans may participate in radical scavenging through their phenolic hydroxyl groups and may exhibit antioxidant activity in some in vitro radical-scavenging and lipid peroxidation inhibition models [11]. Flax lignans have demonstrated protective effects in several animal models of inflammation, particularly regarding anti-inflammatory and tissue-protective activities [12,13,14].

Following the logical sequence of “biosynthesis in plants—processing and metabolic conversion after ingestion—biological activities and applications,” this review summarizes research progress on flax lignans. It first outlines their structural characteristics, distribution, and occurrence forms; then reviews progress in SDG biosynthesis, extraction and purification, analytical detection, and metabolic conversion in vivo; and finally discusses limitations in industrial application and future directions. This review is intended to provide a reference for mechanistic research, quality evaluation, and product development of flax lignans.

## 2. Chemical Structure, Physicochemical Properties, Distribution, and Occurrence Forms of Flaxseed Lignans

### 2.1. Chemical Structure and Physicochemical Properties

Lignans are generally formed through the oxidative coupling of two phenylpropanoid C6–C3 units. The major flaxseed components SDG and SECO are representative dibenzylbutane-type lignans. As the predominant form of flaxseed lignans, SDG is the diglucoside form of SECO. Its phenylpropanoid-derived structural units are connected through β-β′ coupling to form a dibenzylbutane skeleton, and the two glucosyl groups are mainly attached to the side-chain hydroxyl groups at the C9/C9′ positions [15]. This diglucosylated structure is one of the key features that distinguishes SDG from many lipophilic plant lignans; however, plant lignans may also occur as glycosides, ester-linked forms, or polymeric forms [16]. In addition to SDG, small amounts of other lignans or related glycosides/oligomers can be detected in flaxseed, such as SECO and secoisolariciresinol monoglucoside (SMG). Differences in their contents, structures, and activities should still be interpreted in relation to the specific compound and experimental system [5].

**Figure 1:**
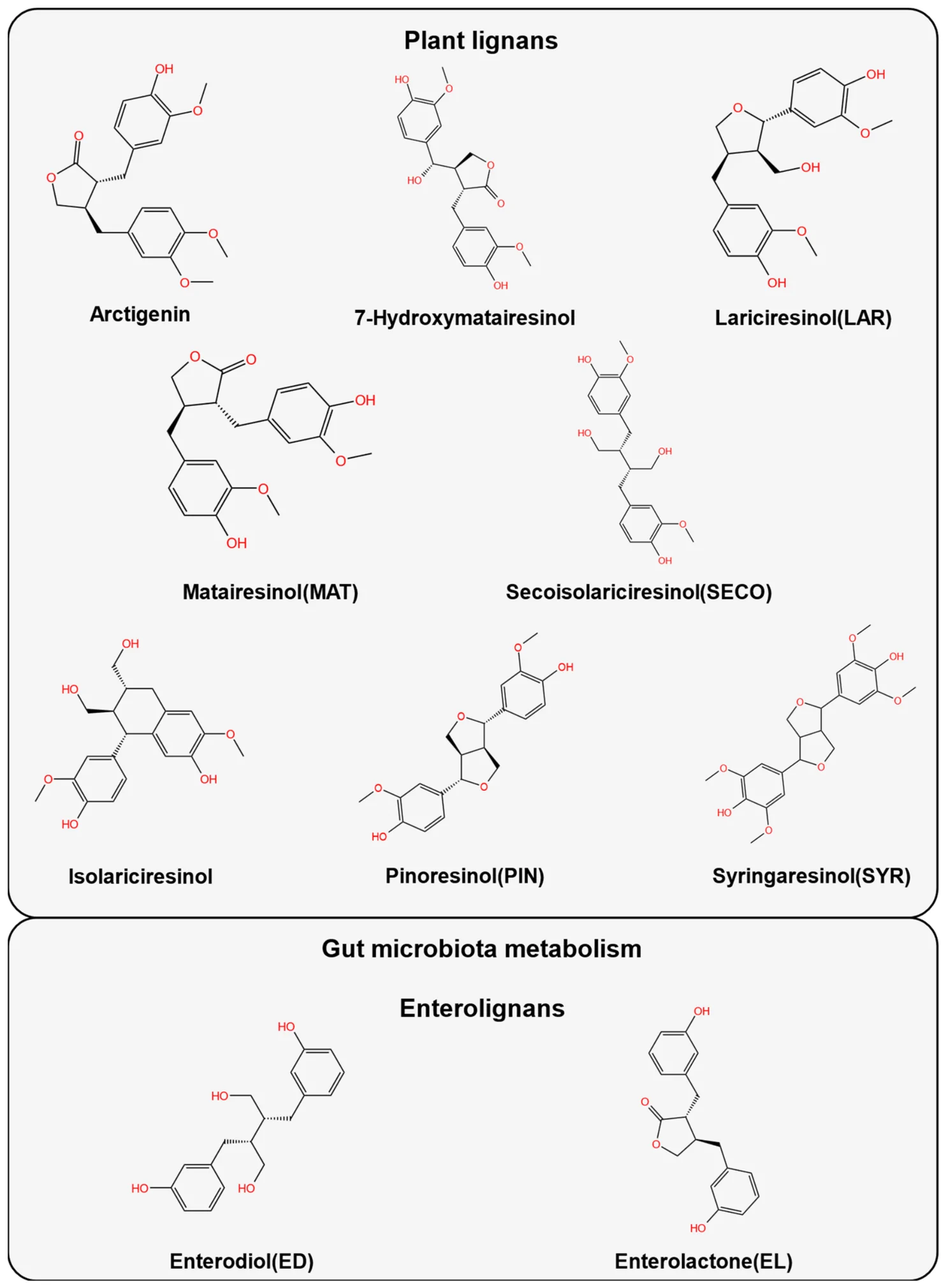
Representative plant lignans and enterolignans.

From the perspectives of dietary intake and human metabolism, lignans can be divided into plant lignans and enterolignans formed by the gut microbiota (Figure 1). Major plant lignans include secoisolariciresinol (SECO), lariciresinol (LAR), isolariciresinol, pinoresinol (PIN), syringaresinol (SYR), matairesinol (MAT), 7-hydroxymatairesinol and arctigenin. After dietary intake, these compounds can be converted by the human gut microbiota into metabolites such as enterolignans [17,18]. The principal enterolignan metabolites include enterodiol (ED) and enterolactone (EL), both of which are considered important metabolic forms of plant lignans in humans and have been associated with potential physiological effects related to antioxidant and anti-inflammatory activity and chronic disease risk [18,19] (Figure 1). The complete process from lignan biosynthesis in plants to metabolic conversion in humans provides the material basis for the nutritional value and physiological functions of flaxseed lignans.

The biological activities of flaxseed lignans are closely related to their chemical structures. Key structural determinants are substituents on the aromatic rings, glycosylation modification and stereochemical configuration [20]. The number and position of phenolic hydroxyl groups, methoxy substitution, glycosylation, and molecular conformation can influence the antioxidant activity of lignans. Studies in the literature have shown that SDG and SECO are antioxidants in models of DNA damage and lipid peroxidation due to 2,2′-Azobis(2-amidinopropane) dihydrochloride (AAPH). Activity differences between SDG and SECO may be related to structural factors such as phenolic hydroxyl groups, methoxy groups, glycosylation status and molecular polarity [21]. Methoxy groups and other structural modifications may alter molecular lipophilicity, membrane permeability and antioxidant features, whereas their contributions to anti-inflammatory and antitumor activities should be evaluated in relation to specific derivatives and experimental models [20]. Glycosylation may increase SDG’s polarity and water solubility and may influence its stability and release in food matrices [5,15]. Stereochemical configuration is also an important structural factor affecting lignan biosynthesis and activity [22]. Studies have shown that flaxseed SDG is mainly derived from SECO with a specific configuration, and its formation process is regulated by the enantioselectivity of enzymes such as pinoresinol–lariciresinol reductase (PLR). In addition, meso-SECO and (+)/(−)-SECO differ in terms of in vitro activities such as immunomodulatory and cytotoxic effects, suggesting that the stereochemical configuration of SECO-type lignans may influence their activity [23,24].

### 2.2. Distribution and Occurrence Forms

Flaxseed lignans, especially SDG, mainly accumulate in seeds, particularly in seed coat-associated tissues. Their levels in vegetative organs are relatively low, although the specific levels are affected by cultivar, developmental stage, and detection method. Tissue localization studies have shown that flaxseed lignans are mainly localized in the seed coat, especially in cell layers associated with the outer integument, whereas little or no accumulation occurs in the embryo [25]. The contents of SDG and total lignans differ markedly among flax lines. Previous studies have indicated that flaxseed SDG content is jointly influenced by genotype, cultivation site, year, and their interactions, with genotype generally being an important determinant [26,27]. The effect of water stress on SDG content is also condition-dependent and may interact with seed coat color and genetic background, thereby affecting lignan accumulation [28]. In addition, SDG accumulation is closely related to seed developmental stage. Developmental studies have shown that SDG and its precursors change dynamically during flaxseed formation and maturation and reach relatively high levels at maturity [25,29].

Lignans in flaxseed occur mainly in free and glycoside-bound forms. The glycoside-bound lignans are the major part of the total lignans, accounting for >90% and mainly existing as SDG [15,30]. Free lignans occur in lower amounts and are more susceptible to oxidative degradation, resulting in poorer stability [16]. SDG in flaxseed is generally present in lignan macromolecular or oligomeric complexes, often complexed through 3-hydroxy-3-methylglutaric acid (HMGA) residues and with phenolic acids such as ferulic acid and *p*-coumaric acid [15]. These complex occurrence forms can influence the release, extraction efficiency, stability and in vitro antioxidant performance of the SDG [31]. Furthermore, the complex flaxseed matrix comprising proteins, polysaccharides and cell wall components may affect the release and extraction efficiency of SDG. Therefore, disruption of the complex structure by crushing, enzymatic hydrolysis, or related methods is required to fully release SDG [32].

Compared with lignans from common dietary sources such as sesame seeds, cereals, and most legumes, flaxseed lignans have advantages in both content and structural characteristics (Table 1) [17]. Among dietary sources, flaxseed is reported to have one of the highest lignan contents and is widely used as plant material for SDG extraction [19] (Table 1). Lignan structural forms vary considerably depending on plant species; sesame seeds predominantly contain lipophilic lignans, whereas flaxseed accumulates glycosylated forms. Lignans in flaxseed are mainly present as SDG, which is characterized by diglucosyl substitution. These structural differences influence solubility, stability, and biological activity [16].

## 3. Regulation of Flax Lignan Biosynthesis in Flax

### 3.1. Biosynthetic Precursors and the Core Pathway of Flax Lignans

The carbon skeleton of SDG in flaxseed is derived from the phenylpropanoid pathway. Its upstream precursor, L-phenylalanine, is generally produced through the plastidial shikimate pathway and then enters cytosolic phenylpropanoid metabolism, thereby supplying precursors for the formation of monolignols and lignan skeletons [5]. L-phenylalanine is deaminated by phenylalanine ammonia-lyase (PAL) to form cinnamic acid, which represents one of the initial reactions through which phenylpropanoid metabolism enters downstream branches such as lignin, flavonoid, and lignan biosynthesis. Changes in PAL expression or activity can influence the metabolic flux towards downstream lignan biosynthesis [4]. Cinnamic acid is then hydroxylated to *p*-coumaric acid by cinnamate 4-hydroxylase (C4H). *p*-coumaric acid is then activated by 4-coumarate:CoA ligase (4CL) to *p*-coumaroyl-CoA. This step is an important link between general phenylpropanoid metabolism and monolignol biosynthesis [35]. Further downstream, *p*-coumaroyl-CoA enters the monolignol biosynthetic branch through reactions catalyzed by hydroxycinnamoyl-CoA shikimate/quinate hydroxycinnamoyltransferase (HCT), *p*-coumaroyl shikimate/quinate 3′-hydroxylase or coumarate 3-hydroxylase (C3′H/C3H), caffeoyl shikimate esterase (CSE), caffeoyl-CoA O-methyltransferase (CCoAOMT), and caffeic acid O-methyltransferase (COMT), leading to the formation of guaiacyl and syringyl monolignol precursors [35]. In this process, cinnamoyl-CoA reductase (CCR) generally reduces the corresponding hydroxycinnamoyl-CoA intermediates to aldehydes, and cinnamyl alcohol dehydrogenase (CAD) further reduces these cinnamaldehyde intermediates to monolignols, including *p*-coumaryl alcohol, coniferyl alcohol, and sinapyl alcohol. For SDG biosynthesis, coniferyl alcohol is the major direct precursor for subsequent lignan skeleton formation [6].

**Figure 2:**
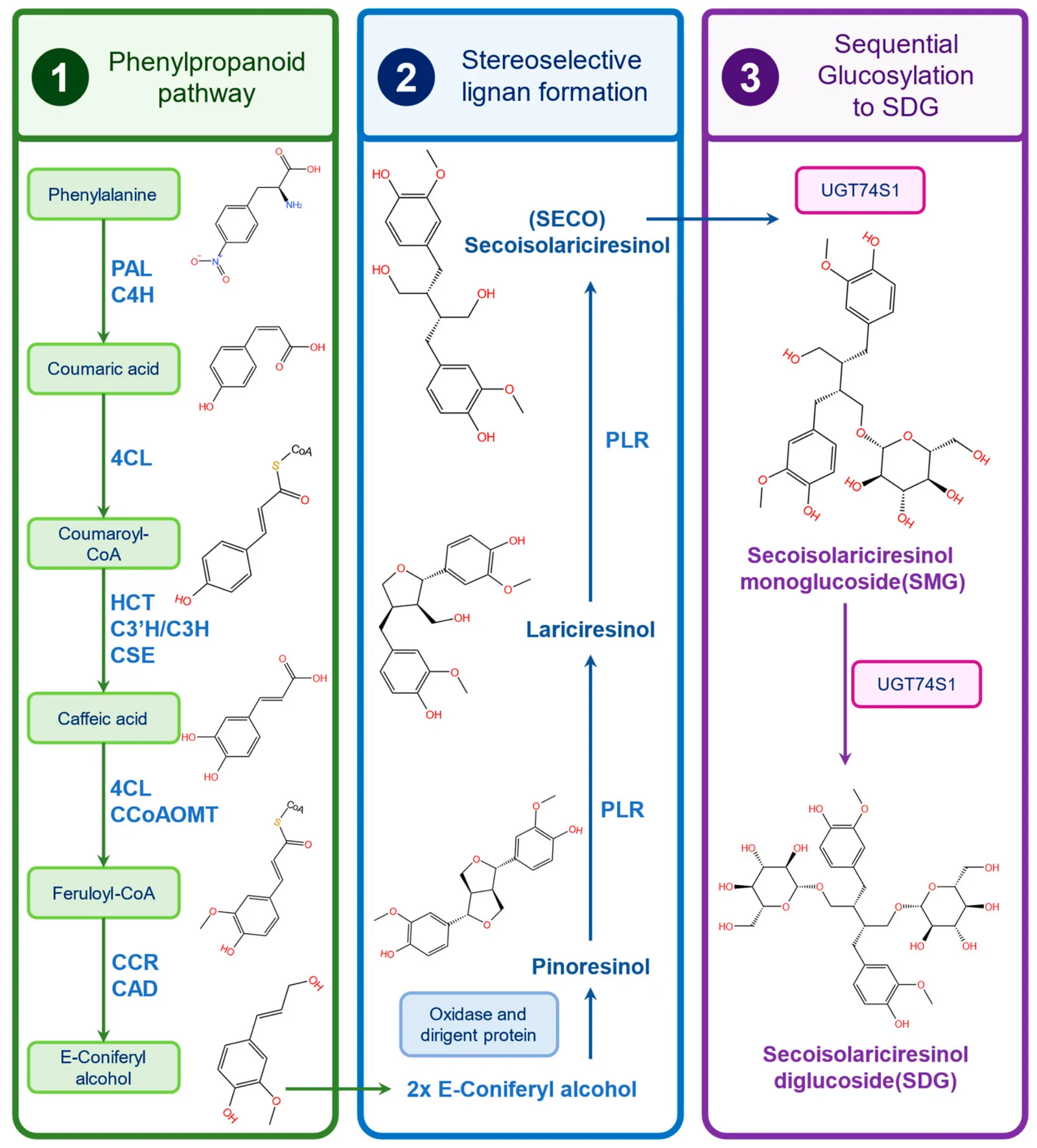
Biosynthetic pathway of secoisolariciresinol diglucoside (SDG).

The core biosynthetic route of flax lignans can be regarded as an extension of phenylalanine metabolism and can be summarized as follows: phenylpropanoid metabolism produces coniferyl alcohol; coniferyl alcohol undergoes oxidative coupling to form pinoresinol; pinoresinol is sequentially reduced by PLR to lariciresinol and SECO; SECO is glycosylated by UDP-glycosyltransferases (UGTs) to form SDG; and SDG is further esterified and assembled into oligomeric complexes [5,36]. First, two molecules of E-coniferyl alcohol undergo stereoselective radical coupling in the presence of oxidases and dirigent proteins (DIR), yielding pinoresinol. PLR then catalyzes the sequential reduction of pinoresinol to lariciresinol and SECO [37]. Therefore, PLR is an important reductase that determines SECO formation and its stereochemical configuration [23]. After SECO is formed, UDP-glycosyltransferases, particularly LuUGT74S1, use UDP-glucose as the sugar donor to sequentially generate SMG and SDG [7,38]. This glycosylation step is mainly associated with the conversion of SECO to SMG and SDG in flax seeds. After SDG formation, SDG can be further assembled through ester bonds with 3-hydroxy-3-methylglutaric acid (HMGA) residues into SDG–HMGA oligomeric complexes, which mainly accumulate in seed coat tissues [36]. In addition, UGT homologs such as UGT74S3 and UGT74S4 may show weaker glycosylation activity toward SECO and can produce small amounts of SMG [7] (Figure 2).

The biosynthesis and accumulation of flax SDG exhibit clear tissue specificity and occur mainly in the seed coat of developing and mature seeds. Laser microdissection and histochemical analyses have shown that SDG biosynthesis and accumulation are primarily localized in the parenchyma layers of the outer integument of the seed coat, whereas the embryo generally does not accumulate SDG or accumulates only very low levels. In terms of spatiotemporal expression, flax SDG biosynthesis is closely associated with seed development. *LuPLR1* has been shown to be expressed in a spatiotemporally specific manner in the seed coat of developing flax seeds, and its expression domain highly overlaps with the sites of SDG accumulation [36]. Based on the preceding analysis, the biosynthesis pathway of flax lignans is regulated by multiple factors, including the development stage, tissue-specific expression, and key enzyme activities, which together form an integrated metabolic network that influences the biosynthesis and accumulation of flax lignans in plants.

### 3.2. Key Biosynthetic Enzymes and Regulatory Mechanisms

Flax SDG biosynthesis consists of several steps, including phenylpropanoid metabolism, oxidative coupling of coniferyl alcohol, dibenzylbutane-type lignan formation and glycosylation [6]. Key enzymes involved in this process are the up-stream phenylpropanoid enzymes, PLR, and the UGTs for glycosylation [35]. Among the enzymes, PLR and UGT74S1 are the most related to SECO and SDG. PAL catalyzes the deamination of L-phenylalanine to cinnamic acid and is one of the initial enzymes of the phenylpropanoid pathway; changes in its expression or activity may influence precursor supply to the lignan biosynthetic branch. C4H participates in the hydroxylation of cinnamic acid to *p*-coumaric acid and may indirectly affect SDG accumulation by altering the supply of phenylpropanoid intermediates. 4CL acts on the carboxyl group of 4-coumaric acid and catalyzes its conjugation with coenzyme A to form 4-coumaroyl-CoA; this enzyme can recognize multiple cinnamic acid derivatives and provide diverse intermediates for downstream metabolism. CCR typically reduces hydroxycinnamoyl-CoA intermediates to the corresponding aldehydes, and CAD further reduces cinnamaldehyde intermediates to monolignols, such as coniferyl alcohol. Coniferyl alcohol then undergoes oxidative coupling to form pinoresinol and enters the lignan biosynthetic branch [39]. PLR is a major reductase in the formation of SECO, and its expression and stereoselectivity are tightly associated with the accumulation of SECO/SDG. More specifically, two molecules of coniferyl alcohol are first stereoselectively coupled in the presence of oxidases and DIRs to yield pinoresinol. Then, pinoresinol is reduced by PLR to lariciresinol and SECO. This enzyme may affect the supply of SECO precursors and also affect the levels of SDG accumulation [37]. UGT74S1 uses UDP-glucose as a sugar donor and catalyzes sequential glycosylation of the C9/C9’ hydroxyl groups of SECO to give SMG and subsequently SDG [8]. Impaired function of this gene has been reported to significantly alter the SMG/SDG accumulation profile and can even result in the absence of SDG [38] (Figure 2).

Flax lignan biosynthesis is regulated by multiple layers, including phytohormone signaling, transcriptional regulation and miRNA-mediated regulation. Among these mechanisms, relatively clear evidence is currently available for the regulatory role of abscisic acid (ABA) in *LuPLR1* expression and SECO/SDG accumulation. ABA can regulate the expression of *LuPLR1* in developing flax seeds and affect the accumulation of SECO, indicating that ABA signaling is involved in the developmental regulation of lignan biosynthesis in the flax seed coat [9]. Corbin et al. further analyzed the *LuPLR1* promoter and found that ABA and gibberellin (GA)-related cis-acting elements participate in the regulation of *LuPLR1* expression and lignan biosynthesis, indicating that phytohormones mainly regulate lignan biosynthetic flux by affecting transcription of key enzyme genes [40]. In addition, Markulin et al. also reported that the Ca^2+^ signaling and calmodulin-like protein LuCML15b modulated the ABA-mediated regulation of flax lignan biosynthesis. This process involves ABRE and MYB2 cis-acting elements in the *LuPLR1* promoter, further indicating that ABA–LuPLR1 regulation has a complex signal-transduction basis [41].

Regarding transcription factors, previous studies have shown that LuWRKY36 can bind to the W-box in the *LuPLR1* promoter and promote *LuPLR1* expression and SECO biosynthesis in a *Fusarium*-induced flax hairy root system. *LuWRKY36* is therefore one of the transcription factors currently supported by experimental evidence in the regulation of flax lignan biosynthesis [10]. In recent years, miRNA-mediated regulation has also been used to dissect the regulatory network of flax lignan biosynthesis. Based on small RNA and degradome data from flax seeds at different developmental stages, Xie et al. constructed a regulatory network involving miRNAs, phasiRNAs, and target genes related to lignan biosynthesis [42]. However, these findings mainly provide candidate regulatory relationships and require further validation through transgenic approaches, mutants, or promoter-binding assays [43].

The accumulation of flax lignans, especially SDG, is jointly affected by genetic background, developmental stage, and environmental conditions. The multi-location study of oilseed flax revealed that SDG content was influenced by both genotype and environment, with genotype and environment accounting for 51.38% and 44.40% of the variation in SDG content, respectively. Altitude may indirectly affect SDG accumulation through integrated ecological factors such as temperature, humidity, and photoperiod [27]. The study by Garros et al. on different cultivars, years, and locations also showed that the contents of flaxseed lignans and related phenolic compounds are jointly influenced by cultivar type, cultivation year, and location, suggesting that SDG accumulation has clear genotype-by-environment interaction characteristics [26]. Experiments on phosphorus fertilizer supply further indicated that year, cultivar, and phosphorus level affect flaxseed lignan yield. Lignan yield increased as phosphorus supply increased from 0 to 80 kg P_2_O_5_ ha^−1^ and then tended to plateau [44].

Water availability is one of the environmental factors for which relatively clear experimental evidence is currently available. Zare et al. found an interaction between water stress and seed coat color. Under non-stressed conditions, yellow-seeded materials had higher SDG content; under water stress, however, SDG content increased in brown-seeded materials and exceeded that of yellow-seeded materials. These findings indicate that the effect of water stress on SDG accumulation depends on genotype and seed coat color [28].

In conclusion, the effects of environmental and agronomic factors on flax lignan accumulation can be explained as a genotype–environment–developmental stage interaction. Future studies should systematically elucidate the impact of environmental factors on the lignan biosynthetic flux in flax by combining multi-location field trials, samplings at defined developmental stages, targeted quantification of SDG/SECO/SMG, assessment of key enzyme activities and expression and functional analyses of key genes.

### 3.3. Research Methods and Technologies for Studying Lignan Biosynthetic Metabolism

The central aim of research on flax lignan biosynthetic metabolism is to clarify the spatiotemporal distribution of SDG and its intermediates in flax tissues, the catalytic steps mediated by key enzymes, and the associated regulatory mechanisms. Compared with earlier approaches that mainly relied on candidate-enzyme inference and product detection, recent studies in this field increasingly use an integrated strategy combining spatial localization, omics-based screening, enzymatic validation, and genetic functional validation, thereby improving the accuracy of pathway reconstruction. First, tissue- and cell-level localization analysis is an important basis for identifying the sites of SDG biosynthesis and accumulation. By integrating genomic, transcriptomic, MALDI-MSI, and bioinformatic analyses, Dalisay et al. investigated the spatiotemporal formation and deposition of lignans and cyanogenic glycosides during flax seed development. The results indicated that the dirigent protein genes related to pinoresinol formation are differentially expressed during developmental stages, and MALDI-MSI can be used to localize SDG and its hydroxymethylglutaryl ester derivatives in seed tissues [39]. Fang et al. subsequently used laser microdissection (LMD) to isolate different seed coat cell layers and combined this approach with nuclear magnetic resonance (NMR), high-performance liquid chromatography (HPLC), and a *LuPLR1* promoter–GUS reporter system. They demonstrated that SDG is mainly synthesized and accumulated in the parenchyma layers of the outer integument of the flax seed coat [36]. Such spatial metabolomics and tissue-specific expression analyses avoid the limitation of inferring biosynthetic sites solely from whole-seed extracts and provide an important technical foundation for elucidating flax lignan biosynthetic metabolism.

For candidate-gene discovery, transcriptome sequencing, full-length transcript sequencing, genome mining, and co-expression analysis can be used to screen enzyme genes and regulatory factors associated with SDG biosynthesis. Flax SDG biosynthesis is closely related to the phenylpropanoid pathway, including the downstream key steps of PLR-mediated reduction and UGT-mediated glycosylation [7,8,23]. In this process, LuPLR1 and UGT74S1 are currently among the most extensively studied core functional genes. Heimendahl et al. demonstrated through heterologous expression and enzymatic assays that flax PLR can convert pinoresinol with specific configurations into the corresponding SECO configuration, suggesting that PLR is one of the key enzymes determining lignan stereoselectivity [23]. Ghose et al. cloned and characterized five flax UGT candidate genes and found that only UGT74S1 uses SECO as a substrate to sequentially generate SMG and SDG, thereby providing key evidence for the SECO glycosylation step in SDG biosynthesis [8]. Fofana et al. further used genome-wide UGT mining, phylogenetic analysis, heterologous expression, and enzyme activity assays to show that the flax genome contains 299 UGTs, of which 241 are duplicated. UGT74S1 is a single-copy gene located on chromosome 7. Although UGT74S3 and UGT74S4 are closely related to UGT74S1, their glycosylation activity toward SECO is approximately seven-fold lower, and they cannot convert SMG to SDG. Therefore, UGT74S1 is considered a key to controlling SDG formation [7]. These studies show that, for flax lignan biosynthetic metabolism, the function of candidate genes cannot be inferred solely from sequence homology; instead, in vitro enzymatic assays and in vivo functional validation are required.

Enzymatic characterization to confirm important biosynthetic steps is usually necessary and involves cloning of candidate genes, heterologous expression, purification of recombinant protein, feeding of substrate and detection of product. For PLR, interconversion among pinoresinol, lariciresinol and SECO could be detected to determine catalytic direction, substrate preference and stereoselectivity. For UGT74S1, SECO, SMG, and UDP-glucose can be used as reaction substrates, and glycosylation products can be confirmed by HPLC or LC–MS/MS [7,8]. In recent years, the catalytic mechanism of UGT74S1 has also been analyzed by structural modeling, molecular docking, and site-directed mutagenesis. Moree et al. performed three-dimensional structural modeling, molecular docking, and enzyme activity analysis of ten *LuUGT74S1* mutants. They found that the S82F and E189L mutations caused complete loss of enzymatic activity, whereas A17 and Q136 played important roles in the conversion of SMG to SDG. Their kinetic results also showed that monoglycosylation is kinetically more favorable, whereas the diglycosylation reaction tends to be irreversible [38]. These findings indicate that enzymatic studies not only confirm key reactions in metabolic pathways but also provide a basis for subsequent protein engineering aimed at increasing target products or regulating the SMG/SDG ratio.

In addition, integrated multi-omics analysis is emerging as a central direction for elucidating the regulatory network of flax lignan biosynthesis. Full-length transcript sequencing and transcriptome analysis can improve the accuracy of gene annotation and help identify shared metabolic nodes among phenylpropanoid metabolism, lignin biosynthesis, and lignan biosynthesis [45]. Combined with metabolomics, spatial mass spectrometry imaging, and enzymatic data, these approaches can further separate candidates that are merely expression-associated from those functionally relevant, thereby reducing false inferences that arise from relying solely on differential expression analysis. Overall, tissue specific localization and metabolite detection should be the backbone for studies of flax lignan biosynthetic metabolism with validated genes such as *LuPLR1* and UGT74S1 as the main references. In the future, a complete research framework of precursor formation, stereoselective reduction, glycosylation modification and environmental and hormonal regulation can be gradually established by integrating transcriptomics, metabolomics, recombinant enzyme assays, RNAi and mutant material analysis.

## 4. Main Biological Activities of Flax Lignans

**Figure 3:**
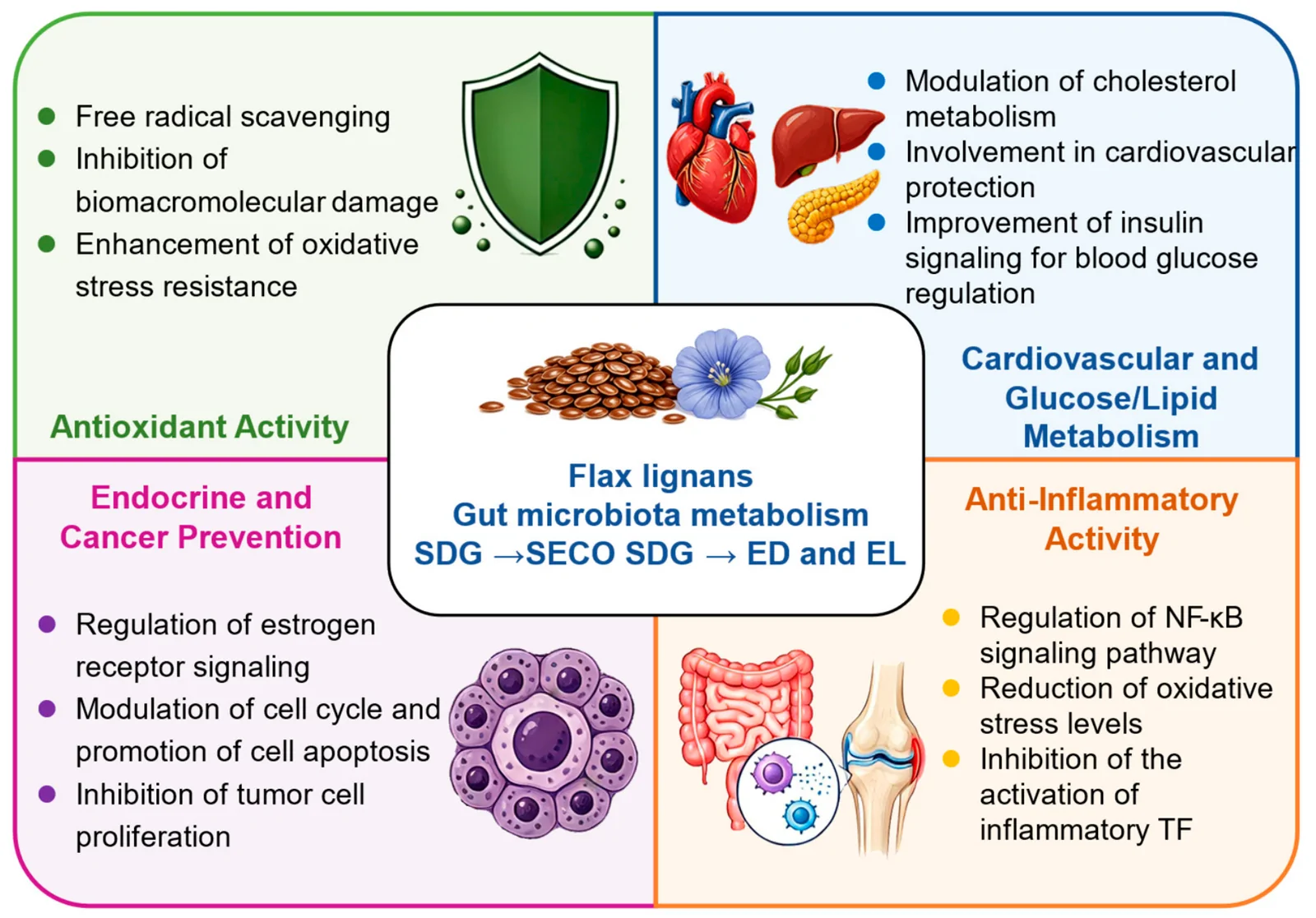
Physiological activity and application of flax lignans.

The biological activities of flax lignans are mainly related to SDG and its metabolites generated by gut microbiota. After ingestion, SDG may be hydrolyzed by gut microbiota to SECO, which may be further converted to enterodiol (ED) and enterolactone (EL). Given the polyphenolic structural features of these metabolites, they potentially could be implicated in metabolic regulation through antioxidant and anti-inflammatory activities, modulation of lipid and glucose metabolism, and actions on hormone-related signaling pathways [18,46,47] (Figure 3).

### 4.1. Antioxidant Activity

Antioxidant activity is one of the most extensively investigated biological activities of flax lignans. SDG, SECO, and their gut-derived metabolites ED and EL all contain phenolic hydroxyl groups. These groups can contribute to radical scavenging through hydrogen- or electron-donating reactions and may, to some extent, inhibit lipid peroxidation and oxidative damage to biological macromolecules. In vitro studies have shown that SDG and its metabolites exhibit antioxidant activity in different radical-scavenging, DNA oxidative damage, and lipid peroxidation models [21,48].

Mechanistically, the antioxidant effects of flax lignans can be summarized into two major pathways. The first is direct chemical antioxidation, which involves phenolic hydroxyl groups that can scavenge free radicals and inhibit the chain reactions of lipid peroxidation, thus reducing the formation of oxidative products such as malondialdehyde (MDA). The second is the regulation of the intracellular antioxidant defense system. Previous studies and reviews have shown that lignan compounds can activate the nuclear factor erythroid 2-related factor 2/antioxidant response element (Nrf2/ARE) pathway and upregulate the antioxidant-related factors, such as heme oxygenase-1 (HO-1), superoxide dismutase (SOD), glutathione peroxidase (GPx), and catalase (CAT), to improve the cellular resistance to oxidative stress [11,49].

### 4.2. Anti-Inflammatory Activity

Anti-inflammatory activities of flax lignans are closely related to their antioxidant activity. The accumulation of reactive oxygen species during inflammatory responses can facilitate the activation of inflammatory signaling pathways such as NF-κB. SDG and its metabolites may exert protective effects by decreasing oxidative stress, inhibiting the activation of inflammatory transcription factors and modulating the expression of inflammatory cytokines [12,13,49]. In a dextran sulfate sodium (DSS)-induced experimental colitis model, SDG ameliorated histopathological injury in colon tissue, inflammatory cell infiltration, and the expression of pro-inflammatory factors. These effects were related to inhibition of NF-κB activation and modulation of the NLRP1 inflammasome. These data suggest that SDG may have tissue-protective effects in models of intestinal inflammation.

Recent experimental studies have even further broadened the potential role of SDG in tissue injury associated with chronic inflammation. SDG can downregulate inflammatory factors and upregulate factors related to cartilage matrix in IL-1β-induced chondrocyte models and animal models of osteoarthritis. These effects are thought to be related to the activation of the Nrf2/HO-1 antioxidant pathway and the inhibition of the NF-κB inflammatory pathway [14].

### 4.3. Endocrine-Related Effects and Potential for Cancer Prevention Research

Flax lignans are relevant plant-derived dietary components, and they are endowed with endocrine-related effects, mainly mediated by ED and EL. ED and EL have partial structural similarity to endogenous estrogens, and they can weakly interact with estrogen receptors, suggesting some regulatory activity [46,50].

Flax lignans have been mainly studied in cancer-related studies, especially in hormone-related tumors, such as breast cancer, and in inflammation-associated model systems. In vitro and animal studies suggest that SDG, SECO, ED and EL may inhibit tumor cell proliferation or tumor growth by affecting estrogen receptor signaling, inhibiting NF-κB activation, regulating the cell cycle, promoting apoptosis and reducing the inflammatory microenvironment. In mouse models of breast cancer, supplementation with SDG led to decreased tumor volume and expression of NF-κB target genes, and its metabolite EL also inhibited breast cancer cell viability and NF-κB signaling in vitro [51]. In an MCF-7 breast cancer xenograft model, flaxseed and purified SDG also showed inhibitory effects on tumor growth [52]. In clinical research, a Phase IIb trial in premenopausal women at high risk of breast cancer used 50 mg/day SDG for 12 months. The results indicated that SDG was generally well tolerated and that reduced Ki-67 levels in benign breast tissue and changes in estrogen receptor-related gene expression were observed [53]. On this basis, current research positions flax lignans as dietary polyphenols with potential value in cancer prevention research; however, further experimental validation and clinical studies are still required.

### 4.4. Cardiovascular and Glucose/Lipid Metabolic Regulation

Cardiometabolic regulation is one of the most widely discussed application directions for flax lignans. Epidemiological studies have shown that higher dietary lignan intake is associated with a lower risk of coronary heart disease [54]. Mechanistically, flax lignans and their metabolites may contribute to cardiovascular protection through antioxidant and anti-inflammatory effects, improvement of endothelial function, effects on cholesterol metabolism and modulation of gut microbiota metabolism [46,55].

Some human intervention studies have hinted that SDG might improve certain lipid-related parameters. Fukumitsu et al. studied men with moderate hypercholesterolemia and found that daily consumption of 100 mg SDG for 12 weeks reduced the low-density lipoprotein cholesterol/high-density lipoprotein cholesterol (LDL-C/HDL-C) ratio and improved some liver function-related risk indicators [56]. Another clinical study showed that, among individuals with borderline elevated LDL-C, daily intake of 60 mg SDG for 12 weeks reduced LDL-C and total cholesterol in male participants [57]. These clinical studies indicate that SDG may show favorable trends in LDL-C, total cholesterol, or the LDL-C/HDL-C ratio. However, sex differences and study design are limited by the sample size, and thus, further clinical studies are needed.

In terms of glucose metabolism, flax lignans may affect glycemic control through enhancement of oxidative stress, inflammatory status and insulin signal transduction. In animal experiments, SDG or flaxseed lignan-enriched fractions improved blood glucose, lipid metabolism and antioxidant status in diabetic models [58]. In a human clinical study, 73 patients with type 2 diabetes and mild hypercholesterolemia received a flaxseed lignan supplement containing 360 mg/day SDG for 12 weeks. Compared with placebo, lignan supplementation produced a small decrease in glycated hemoglobin (HbA1c), suggesting a possible improvement in glycemic control [59].

Regarding lipid metabolic regulation, a recent animal study showed that, in non-fasted mice, co-ingestion of flax lignans increased serum eicosapentaenoic acid (EPA) and docosahexaenoic acid (DHA) levels by 31.9% and 20.2%, respectively, compared with intake of flaxseed oil nanoemulsion alone. Co-ingestion also increased hepatic EPA levels by 35.1%. SDG and SECO showed different degrees of promotion of the conversion of α-linolenic acid (ALA) into n-3 long-chain polyunsaturated fatty acids (n-3 LC-PUFAs). These findings indicate that flax lignan intake can affect the absorption, transport, and hepatic metabolism of ALA, providing a new research strategy for improving the utilization efficiency of plant-derived ALA [60].

In summary, the main biological activities of flax lignans comprise antioxidant and anti-inflammatory effects, basic research of cancer prevention, and regulation of cardiovascular and glucose/lipid metabolism. Future studies should further distinguish the functional differences among different active forms, including SDG, SECO, ED, and EL, and should integrate gut microbiota-mediated metabolic capacity, dose–response relationships, and population stratification into systematic evaluation and clinical validation.

## 5. Current Status, Challenges, and Research Gaps in Industrial Application

In vitro studies, animal experiments and human studies suggest that flax lignans and their metabolites derived from gut microbiota may have multiple biological activities, such as antioxidant and anti-inflammatory effects, cardiometabolic regulation and potential regulation of risks of hormone-related diseases. SDG and the fractions enriched in flax lignans have the potential to be used in the development of functional foods and dietary supplements [46,61]. In pharmaceutical and nutritional intervention research, flax lignans may be candidate adjunctive dietary components for chronic disease risk-management studies, in particular for those focused on hormone-associated tumors such as breast cancer, cardiometabolic abnormalities and glucose/lipid metabolic disorders [5]. In the fields of food, nutrition, and health, they can be used as dietary lignan sources or functional food ingredients for products related to antioxidant activity and cardiometabolic health [61,62]. In cosmetics, their antioxidant properties also suggest exploratory value in products related to skin oxidative stress. Flaxseed extracts and their lignan components have already been studied in formulations related to antioxidant skin care and improvement of skin condition [63]. With the development of functional foods and research on natural bioactive compounds, the extraction, stabilization, and translational application of flax lignans continue to receive attention.

Among these application areas, functional foods and dietary supplements represent the field closest to market realization for the following reasons: (1) the regulatory pathway for dietary supplements is generally less stringent than for pharmaceuticals; (2) existing clinical evidence, while limited, supports the safety and potential efficacy of SDG-containing supplements; and (3) consumer demand for plant-derived bioactive compounds continues to grow. In contrast, pharmaceutical applications remain at earlier stages due to the need for more rigorous clinical validation, while cosmetic applications are still largely exploratory.

**Figure 4:**
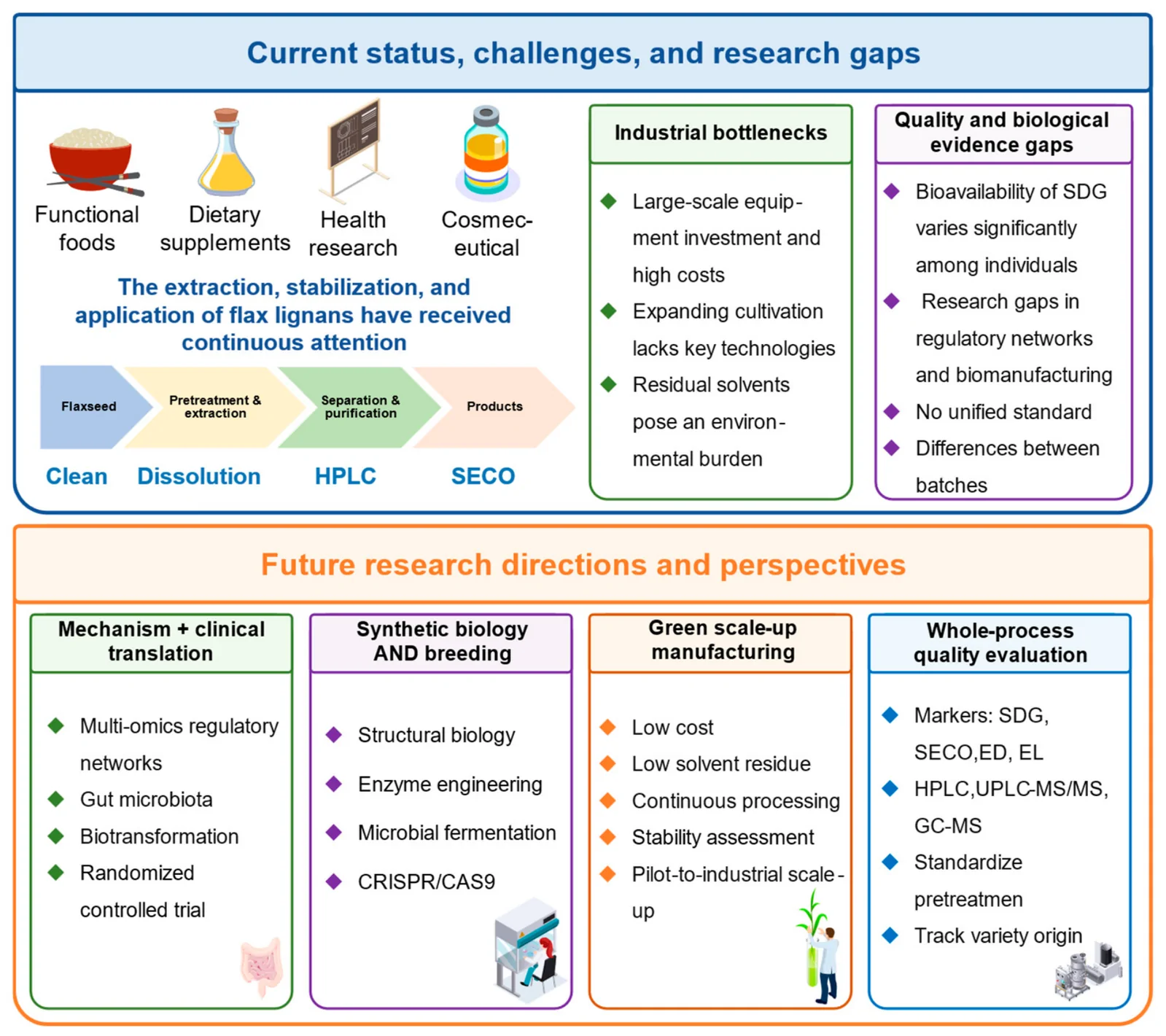
Industrial application status, challenges, research gaps, and future perspectives of flax lignans.

While industrial application of flax lignans has advanced, challenges remain in large-scale and high-quality development (Figure 4). Flax lignans are typically isolated and concentrated from defatted flaxseed meal, flaxseed hulls or flaxseed cake. Common methods include extraction with aqueous alcohol systems, alkali/alcoholysis-assisted release, ultrasound- or microwave-assisted extraction, pressurized low-polarity water extraction, and supercritical CO_2_ extraction [30,64]. For separation and purification, liquid–liquid partitioning, anion-exchange resin, C18 resin, preparative high-performance liquid chromatography (preparative HPLC), high-speed counter-current chromatography (HSCCC), and column chromatography have all been used to prepare SDG or flax lignan-enriched products. Some studies have obtained SDG with a purity above 98% [65]. However, existing methods still face many problems in scale-up, such as complex hydrolysis and separation procedures, high solvent consumption, high costs of resins or chromatographic materials, and sensitivity of SDG yield and stability to processing conditions [30,66]. These problems lead to substantial equipment investment and high production costs, thereby increasing product market prices and limiting broader application in the food and cosmetics sectors.

The product quality evaluation system is another important factor limiting the industrial application of flax lignans. Current flax lignan products on the market may exist in multiple forms, including flaxseed powder, flaxseed hull extract, SDG-enriched products, SECO, or total lignan complexes. These products can differ substantially in raw material source, pretreatment method, extraction and purification process, SDG content, accompanying polyphenol composition, residual solvents, heavy metals, pesticide residues, and microbiological indicators [67]. Although HPLC and liquid chromatography–tandem mass spectrometry (LC–MS/MS) have been used for the quantitative analysis of SDG and its metabolites [68], these methods are mainly applied to laboratory testing and small-scale sample analysis, and a unified quality standard system has not yet been established. As a result, batch-to-batch quality variation remains considerable, affecting consumer trust and the standardized development of the industry.

From a regulatory perspective, flax lignan products face different requirements depending on the application context and target market. In the United States, SDG-containing products are primarily marketed as dietary supplements under the Dietary Supplement Health and Education Act (DSHEA), which requires pre-market notification but not pre-market approval. In the European Union, flax lignan extracts may be classified as novel foods under Regulation (EU) 2015/2283, requiring pre-market authorization. In China, SDG-related products are regulated as health foods or food additives, with specific requirements for safety evaluation and functional claims. These regulatory differences create challenges for global product development and standardization and highlight the need for harmonized quality standards and safety assessments.

Current experimental research shows that, after ingestion, flax lignans are hydrolyzed by gut microbial β-glucosidases to generate SECO and are then converted through multiple reactions into enterolignans. These compounds are considered important active forms through which lignans exert some weak estrogen-like, antioxidant, and metabolic regulatory effects in humans [46]. From the perspectives of bioavailability and precision application, the in vivo metabolic mechanisms of flax lignans remain incompletely elucidated. Individual differences in gut microbiota composition, dietary structure, antibiotic use, age, and metabolic status may lead to variation in flax lignan bioavailability and efficacy [18]. Further clarification is needed regarding the specific microbial taxa involved, key enzyme systems, and cooperative metabolic relationships among microbial communities. Additionally, individual differences in the absorption, conjugated metabolism, tissue distribution, and excretion of enterolignans require systematic investigation. In clinical research, related data remain insufficient, making it difficult to fully verify their bioactivity, safety, and long-term effects in humans. In particular, the lack of sufficient clinical evidence in pharmaceutical contexts limits their broader application [46,50].

Regarding safety considerations, while flax lignans are generally recognized as safe for most populations, several caveats warrant attention. First, the weak estrogenic activity of enterolignans (ED and EL) raises concerns for individuals with hormone-sensitive conditions, such as estrogen receptor-positive breast cancer, although current evidence does not establish a clear risk. Second, the variability in gut microbiota composition means that individual responses to flax lignan supplementation may differ substantially, complicating dose–response relationships. Therefore, while flax lignans show promise as functional ingredients, personalized approaches considering individual health status, medication use, and gut microbiome composition may be necessary for optimal application.

From the perspective of synthetic biology, research on flax lignan biosynthesis and metabolism still has several key gaps. To date, only the functions of a limited number of key enzymes and transcription factors have been clarified [7,8]. Several aspects of the regulatory network remain insufficiently understood, including cooperative mechanisms among key enzymes, interactions between transcription factors and plant hormones, and the effects of environmental factors [27]. Although crystal structures, active sites, and substrate specificities of some key enzymes in the flax lignan biosynthetic pathway have been reported in recent years [67], systematic structure-function studies remain insufficient. On the other hand, SDG production still mainly relies on natural extraction and purification from flaxseed or flaxseed hulls, and efficient heterologous production systems based on engineered microorganisms are not yet mature. Molecular breeding for high-lignan flax varieties also faces challenges such as stable transformation and editing efficiency [69,70,71,72]. These gaps underscore the demand for targeted, systematic strategies across enzyme engineering, heterologous biosynthesis, and molecular breeding, as detailed in Section 6.

## 6. Future Research Directions and Perspectives

In response to the issues discussed above, future research on flax lignans should gradually shift from activity discovery toward a systematic stage integrating mechanism elucidation, quality control, industrial scale-up, and precision application. Research can be advanced in the following four directions (Figure 4).

Multi-omics technologies should be integrated to construct system-level regulatory network models, screen and identify key regulatory nodes, and elucidate the interactions between environmental factors and endogenous regulators. Research should also be strengthened on gut microbiota-mediated biotransformation mechanisms and human clinical evidence. By using ED and EL as biomarkers of human exposure and biological effects, future studies can identify key microbial strains, key enzyme systems, and cooperative relationships among microbial communities; evaluate the biological effects of different lignan metabolites; and clarify their conversion mechanisms. Multicenter, randomized, double-blind, placebo-controlled studies should be conducted to determine the effects of different doses, product forms, and intake durations on blood lipids, blood glucose, inflammation, oxidative stress, hormone-related indicators, and safety.

To address the aforementioned issues, we propose the following strategies. For enzyme engineering, structural biology studies should be integrated with site-directed mutagenesis and directed evolution to improve the catalytic activity and substrate selectivity of PLR and UGTs. For heterologous production, synthetic biology approaches such as metabolic pathway reconstruction in engineered microorganisms (*Saccharomyces cerevisiae* and *Escherichia coli*) should be further developed to achieve efficient, stable, and low-cost fermentative production of SDG and its precursors. For molecular breeding, CRISPR/Cas9 gene editing and genetic transformation can be applied to develop flax varieties with high SDG content, reduced antinutritional factors, and suitability for mechanized processing. These integrated strategies can collectively reduce reliance on natural extraction and facilitate industrial-scale production.

Extraction and preparation technologies should be oriented toward low cost, low solvent residue, continuous processing, and green manufacturing. The extraction and enrichment processes for flax lignans should be advanced from laboratory research toward pilot-scale and industrial production. Relevant studies should not focus solely on improving SDG yield and purity but should also comprehensively evaluate solvent consumption, energy consumption, environmental impact, batch stability, residue control, production cost, and feasibility of scale-up. On this basis, an industrial production process system should be established while ensuring product quality and safety.

A whole-process quality evaluation system covering raw materials, intermediates, and final products should be constructed. In future quality control, SDG, SECO, ED, and EL can be used as core quality markers, combined with technologies such as HPLC, ultra-performance liquid chromatography–tandem mass spectrometry (UPLC–MS/MS), gas chromatography–mass spectrometry (GC–MS), and chemical fingerprinting. Sample pretreatment, reference standard calibration, method validation, and interlaboratory comparison procedures should be standardized. At the same time, raw material origin, variety, seed hull proportion, extraction process, residual solvents, heavy metals, pesticide residues, microbiological limits, stability, packaging and storage conditions, and boundaries for functional claims should be included in the evaluation system, thereby providing quality assurance for standardized applications in functional foods, nutritional supplements, and cosmetic raw materials.

Overall, if high-lignan flax varieties, standardized extracts, gut microbiota stratification, clinical endpoint validation, and a complete quality evaluation system can be integrated, flax lignans may move beyond their current role as research targets for functional ingredients and further develop into important natural bioactive sources for food and nutrition, auxiliary intervention strategies for chronic disease risk management, and products related to skin oxidative stress.

## Figures and Tables

**Table 1:** Comparison of lignan content and major lignan forms in flaxseed and other common dietary sources.

Category	Food/Sample	Measurement Basis μg/100 g	SECO ^a^	MAT ^a^	LAR ^a^	PIN ^a^	SYR ^a^	Ref.
Oilseeds and Nuts	Flaxseed	fresh edible weight	294,210	553	3041	3324	—	[2]
	Flaxseed	wet weight	375,321.90	153.3	2807.50	729.6	—	[3]
	Flaxseed/linseed	dry weight	369,900	1087	—	—	—	[33]
	Sesame seed	fresh edible weight	66	481	9470	29,331	—	[2]
	Sunflower seed	wet weight	26.2	0.5	149.7	33.9	—	[3]
Vegetables	Garlic	fresh edible weight	50	0	286	200	—	[2]
	Garlic/Ninniku	wet basis	55	—	84	45	—	[34]
	Curly kale	fresh edible weight	19	12	599	1691	—	[2]
	Broccoli	fresh edible weight	38	0	972	315	—	[2]
	Asparagus	wet basis	743	14	92	122	58	[34]
	Edible burdock/Gobo	wet basis	251	39	95	74	9	[34]
Fruits	Apricot	fresh edible weight	31	0	105	314	—	[2]
	Strawberry	fresh edible weight	5	0	117	212	—	[2]
	Peach	fresh edible weight	27	0	80	186	—	[2]
	Yuzu	wet basis	26	—	192	654	293	[34]
	Valencia orange	wet basis	56	—	193	51	202	[34]
	Navel orange	wet basis	14	—	128	24	48	[34]
Cereal and Grain	Whole grain flaxseed bread	fresh edible weight	11,845	26	220	383	—	[2]
	Bread, flax	wet weight	7208.30	0.2	29.2	1.6	—	[3]
	Rye bread, 50% rye	wet basis	33	4	47	44	402	[34]

^a^ SECO, Secoisolariciresinol; MAT, Matairesinol; LAR, Lariciresinol; PIN, Pinoresinol; SYR, Syringaresinol.

## Data Availability

No new data were created or analyzed in this study. Data sharing is not applicable to this article.
